# Xpert HIV-1 qual point-of-care testing for HIV early infant diagnosis in Tanzania: experiences and perceptions of health care workers in a 2016 study

**DOI:** 10.1186/s12981-024-00619-2

**Published:** 2024-05-16

**Authors:** Siriel Boniface, Anange Lwilla, Hellen Mahiga, Doreen Pamba, Otto Geisenberger, John France, Rebecca Mokeha, Lilian Njovu, Abisai Kisinda, Nyanda Elias Ntinginya, Michael Hoelscher, Arne Kroidl, Issa Sabi

**Affiliations:** 1 National Institute for Medical Research, Mbeya Medical Research Center, P.O.Box 2410, Mbeya, Tanzania; 2 Division of Infectious Diseases and Tropical Medicine, University Hospital, LMU Munich, Munich, Germany; 3 German Center for Infection Research (DZIF), Munich, Germany; 4 Department of Obstetrics and Gynaecology, Mbeya Zonal Referral Hospital, Mbeya, Tanzania; 5 CIHLMU Center for International Health, University Hospital, LMU Munich, Munich, Germany

**Keywords:** HIV, Early infant diagnosis, Point-of-care testing, Vertical HIV transmission, Primary healthcare workers

## Abstract

**Background:**

HIV early infant diagnosis (HEID) at the centralized laboratory faces many challenges that impact the cascade of timely HEID. Point of Care (PoC) HEID has shown to reduce test turnaround times, allow for task shifting and has the potential to reduce infant mortality. We aimed at assessing the feasibility of nurse based PoC-HEID in five facilities of Mbeya region.

**Methods:**

We analysed data from healthcare workers at five obstetric health facilities that participated in the BABY study which enrolled mothers living with HIV and their HIV exposed infants who were followed up until 6 weeks post-delivery. Nurses and laboratory personnel were trained and performed HEID procedures using the Xpert HIV-1 Qual PoC systems. Involved personnel were interviewed on feasibility, knowledge and competency of procedures and overall impression of the use of HIV-1 Qual PoC system in clinical settings.

**Results:**

A total of 28 health care workers (HCWs) who participated in the study between 2014 and 2016 were interviewed, 23 being nurses, 1 clinical officer, 1 lab scientist and 3 lab technicians The median age was 39.5 years. Majority of the nurses (22/24) and all lab staff were confident using Gene Xpert PoC test after being trained. None of them rated Gene Xpert handling as too complicated despite minor challenges. Five HCWs (5/24) reported power cut as the most often occurring problem. As an overall impression, all interviewees agreed on PoC HEID to be used in clinical settings however, about half of them (11/24) indicated that the PoC-HEID procedures add a burden onto their routine workload.

**Conclusion:**

Overall, health care workers in our study demonstrated very good perceptions and experiences of using PoC HEID. Efforts should be invested on quality training, targeted task distribution at the clinics, continual supportive supervision and power back up mechanisms to make the wide-scale adoption of nurse based PoC HEID testing a possibility.

**Supplementary Information:**

The online version contains supplementary material available at 10.1186/s12981-024-00619-2.

## Background

Despite the tremendous decline of infant HIV infection globally, the reported vertical transmission in Tanzania was estimated at 10.9% in 2021, higher than the global targets of 5% [1]. The program has been successful in ensuring that almost all (92%) pregnant women living with HIV are enrolled in prevention of vertical HIV transmission (PVHT) services, but loss to follow-up during pregnancy and postnatal period remain high. In 2021, it was estimated that only 47.6% of HIV exposed new-borns had a nucleic acid testing (NAT) performed within the recommended two months of life [1]. The Tanzanian Ministry of health adopted the WHO PVHT recommendations which also includes the initiation of enhanced postnatal prophylaxis (ePNP) in infants at high risk of acquiring HIV infection from their mothers. The ePNP consists of either daily zidovudine and nevirapine, or daily zidovudine, lamivudine and nevirapine for the first six weeks of life, and thereafter nevirapine alone up to 12 weeks of life [2]. The guidelines further emphasize viral load monitoring for the mothers during pregnancy and delivery.

The traditional approach involving sample collection by Dried Blood Spot (DBS), and sample transportation for HIV virological testing at the centralized laboratory, is associated with high turnaround times of results often leads to delays of ART initiation [3–5]. Point of care technologies have the potential to overcome challenges associated with centralized laboratory testing by reducing test turnaround times, enabling early ART initiation, and may facilitate task shifting to nurses or minimally trained laboratory personnel to directly perform testing procedures at PoC [6]. Different studies have shown PoC platforms results in improvement in turn around times to as low as 0 days regardless of the type of test used, age of infant or health care facility [4, 5], and takes less than 2 h from sample collection to ART initiation in facilities using PoC testing [7–9].

The Xpert HIV-1 qual (Cepheid Inc., Sunnyvale, CA, USA) was endorsed by WHO for HIV NAT in infant below 18 months to complement the existing conventional high throughput platforms at the centralized laboratory [5]. The Xpert HIV-1 qual test is designed as simple to use suitable for operators with limited experience and training. The maintenance requirements are simple including the basic cleaning procedures and annual calibration check which can be performed by the end user [10, 11]. The test requires a direct, unprocessed whole blood specimen to be added to the cartridge. Dried blood spot (DBS) samples can also be used but this requires a pre analytical incubation of the DBS, in a pre aliquoted sample reagent buffer at 56 °C in a thermomixer for 15 min. We previously reported excellent Xpert HIV-1 Qual performance and good operational feasibility of the PoC-HEID testing at primary health facilities in terms of turn-around-times, error rates and communication of results to the mothers [9]. As an important component of operational feasibility, in this study we went further into assessing the perceptions and experiences on utilization of Xpert HIV-1 qual for HIV EID among health care workers in primary health care settings in Tanzania. These health care workers were those directly involved with performing the tests.

## Methodology

### Study design and population

This study was a cross-sectional descriptive analysis nested into a larger study (the BABY study) which was a prospective diagnostic study evaluating the accuracy and operational characteristics of the Xpert HIV-1 qual PoC test for infants born from mothers living with HIV. The BABY study enrolled 606 mothers living with HIV and their HIV exposed babies at birth and followed up until 6 weeks of age. Infant HIV testing was performed at the time of delivery and thereafter at week 1, 2, 3, and 6 post-delivery using fresh 100 µL heel prick blood for Xpert-PoC test. All tests were performed by trained nurses at respective health facilities. These nurses were employed at their clinics and study procedures were performed in addition to routine clinical procedures. Additional work burden derived through study procedures were compensated by visit with a fixed amount of TZS 12,000/=. At each time point, Dried blood Spot (DBS) samples were collected for qualitative HIV-DNA confirmation as previously described; additional spot from the collected DBS was tested for Xpert HIV-1 qual on the GeneXpert platforms by the laboratory technician at NIMR laboratory. NIMR laboratory was also used as back laboratory, such as during the time of prolonged power cut at the health facilities, therefore laboratory staff were occasionally involved in Xpert HIV-1 qual testing from fresh 100 µL heel prick blood. The required annual calibration checks of the GeneXpert systems were performed by either end user or Cepheid service personnel. Further details on participant recruitment, study procedures, and ethical approvals were previously reported [9].

### Study sites and training

The study was conducted at five public health facilities and Mbeya Medical Research Centre in Mbeya region, Tanzania from 2014 to 2016. The sites included one tertiary level clinic—the maternity wing of Mbeya Zonal Referral Hospital, one secondary level clinic (Mbeya Regional Referral Hospital) and three primary health care facilities (Kiwanjampaka, Ruanda and Igawilo). A two days centralized training was performed, and end users of varying skill level, nurse midwives and lab technicians, were trained according to standard Cepheid procedures and training material. Overall, 24 clinical staff (at the health facilities) and 4 laboratory staff (at NIMR lab) were involved in this training (Table 1). A retraining was conducted during site initiation as well as several on job trainings as the study progressed. At the end of the training, an English written Xpert user SOP was provided for each site at the clinic and laboratory rooms. Operating procedures was also summarized as a poster and placed on a wall for quick referencing.

**Table 1:** Characteristics of study sites and health care workers involved

	MZRH	MRRH	Kiwanjampaka	Ruanda	Igawilo	MMRC
HCWs trained						
Nurse (certificate)	2	2	–	2	1	–
Nurse (diploma)	3	4	1	3	4	–
Nurse (degree)	–	–	1	–	–	–
Clinical Officer	–	–	1	–	–	–
Laboratory Scientist	–	–	–	–	–	1
Laboratory Technician	–	–	–	–	–	3
Total HCWs trained	5	6	3	5	5	4
Median age (range)	45 (37–48)	39 (25–45)	45 (37–56)	39 (38–58)	42 (25–60)	38.5 (33–42)
Facility level	Tertiary	Secondary	Primary	Primary	Primary	Research centre
Power Backup	Yes	Yes	No	No	No	Yes
# infants enrolled per facility	198	80	52	126	158	N/A
# of overall Xpert HIV-1 tests performed**	852	363	248	536	737	974*

HCWs: Health Care Workers; MZRH: Mbeya Zonal Referral Hospital; MRRH: Mbeya Regional Referral Hospital

*(Xpert Qual 379, Xpert Quant 595)

**Numbers are derived from available information in the database and does not include additional tests for training purposes or double testing

### Questionnaire and analysis

At the end of the study in 2016, we interviewed health care workers who participated in Xpert HIV-1 diagnostic testing using a structured questionnaire with questions on a likert scale. Interviewers asked the health care workers questions on perception on quality of training, Gene-Xpert user manual, Gene-Xpert handling and overall expression on performing these tests. (Additional file 1: Appendix 1) Data was captured and analysed using Microsoft Excel software. Only descriptive data analysis was performed and did not include statistical outcomes. In general, responses between clinical and laboratory staff members were stratified, as their qualifications and experiences with respect to the Xpert HIV-1 testing may have been different.

## Results

The BABY study was conducted in the years 2014–2016. A total of 28 health care workers (HCWs) were interviewed where, 23 were nurses, 1 clinical officer, 1 laboratory scientist and 3 laboratory technicians. The median age (range) was 39.5 years (26–60). A total of 606 mothers with 614 infants were included with 2736 HIV-1 tests performed. All included health facilities were affiliated with HIV Care-and-Treatment (CTC) at site except NIMR research laboratory which is a research centre (Table 1).

### Training

**Fig. 1:**
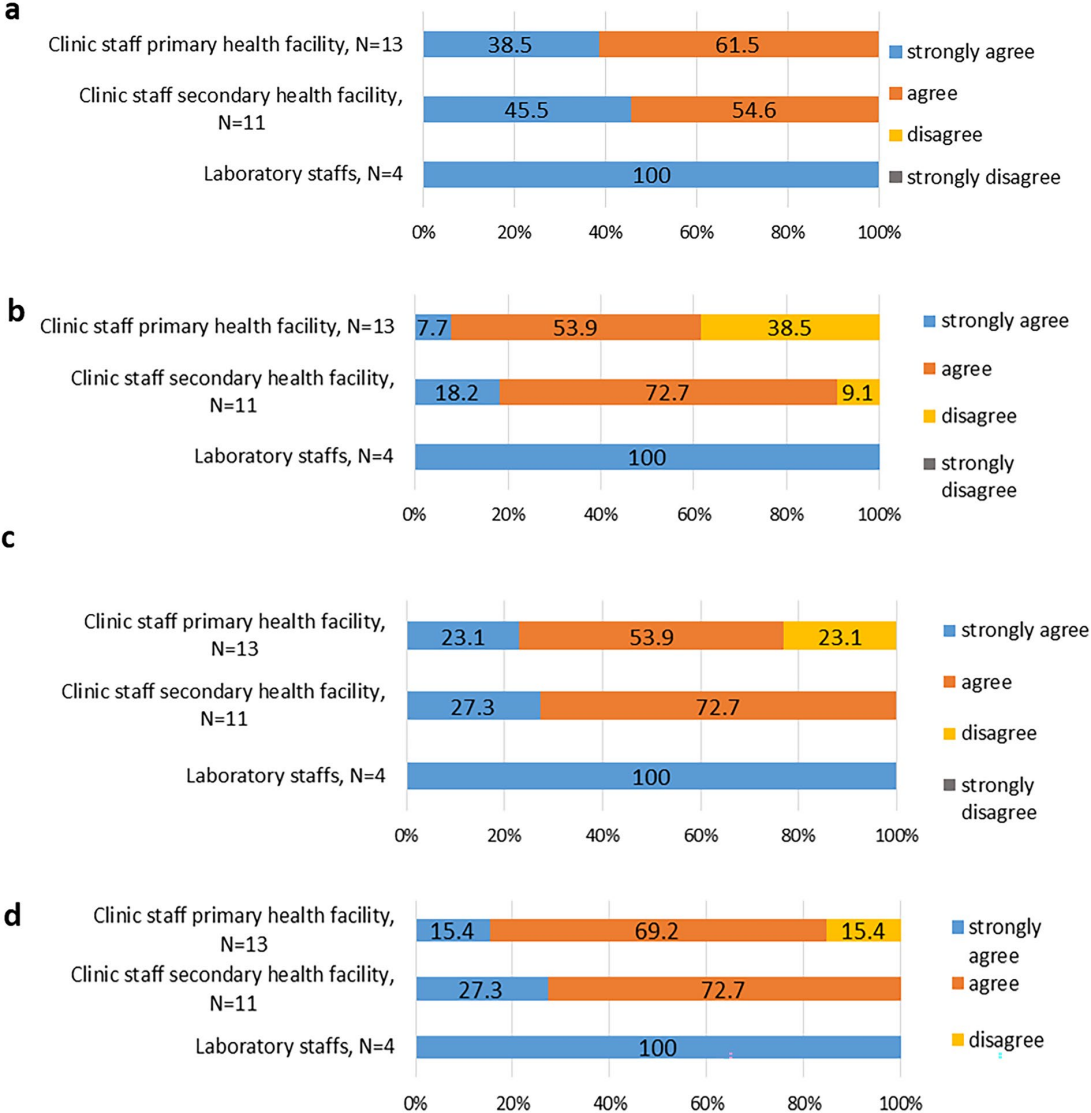
Perception of the level of training provided to health care workers at study initiation. **a** Perception on adequacy of the level of Xpert PoC test training on knowledge and abilities **b** Perception on the adequacy of training time in acquiring intended skills. **c** Perception on adequacy of opportunities to run Xpert test samples. **d** Perception on level of confidence in using Xpert PoC test after the training

All 28 interviewed staff agreed that the level of Xpert PoC training was adequate to their prior knowledge and abilities. All four laboratory staff strongly agreed to this. Among the clinic staff, five (5/13) from primary and five (5/11) from secondary health facilities strongly agreed to having obtained an adequate training (Fig. 1A). While all laboratory staff strongly agreed to have had enough time acquiring the intended skills, only one (1/13) and two (2/11) of clinic staff from primary and secondary health facilities respectively strongly agreed that they had enough time to acquire the intended skills (Fig. 1B). Almost all (21/24) of the clinic staff agreed or strongly agreed to having had enough opportunity to run Xpert test samples for practical training except three (23.1%). These were both from a primary health facility (Fig. 1C). All but two (22/24) of the nurses agreed to having felt confident using the Xpert PoC test after training. The two that did not feel confident after the training were from primary health facilities (Fig. 1D).

### Xpert user manual

**Fig. 2:**
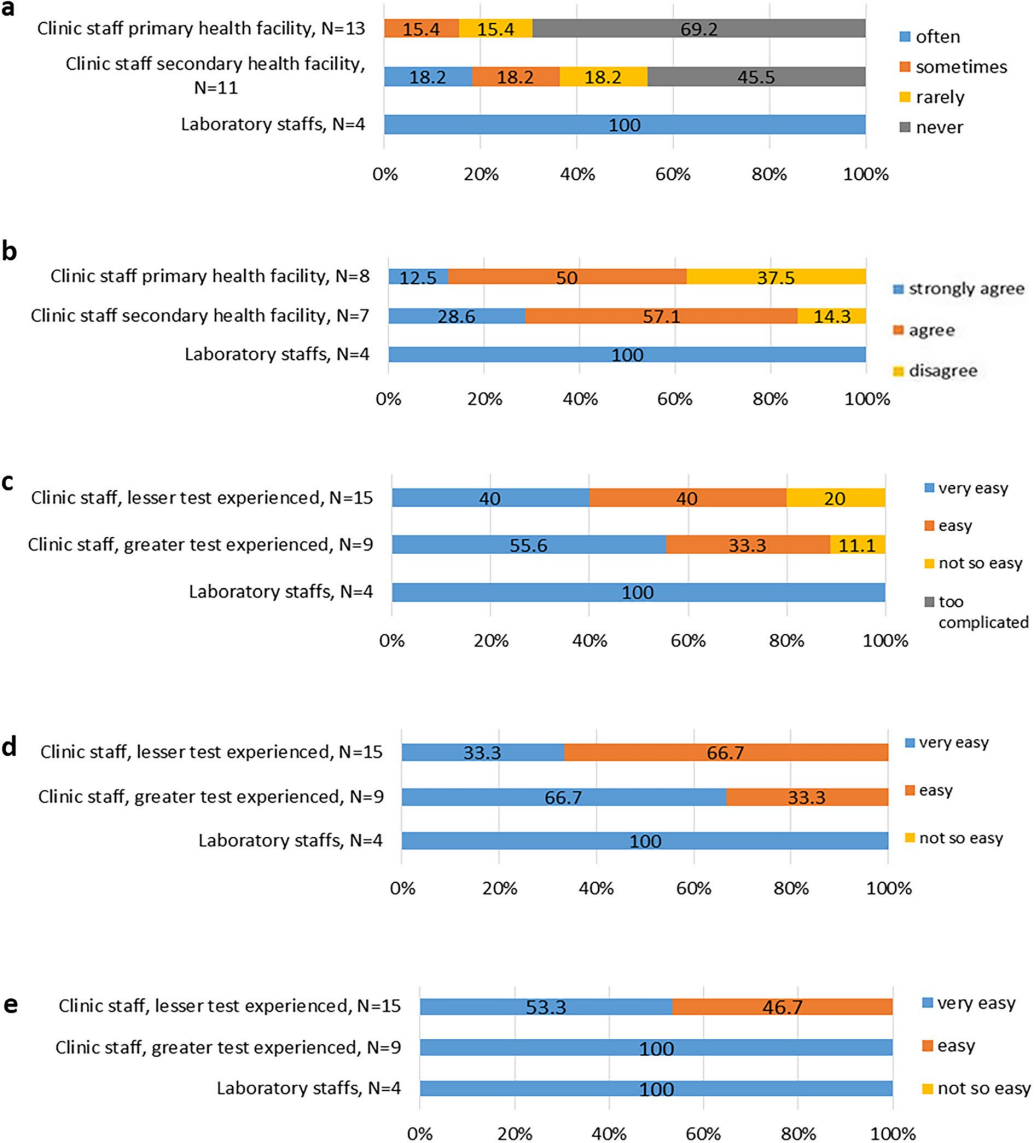
Use of the user manual and handling of the Xpert HIV-1 qual assay by health care workers. **a** Response on the frequency of using the GeneXpert user manual. **b** Perception on how easy it was to understand the GeneXpert user manual. **c** Rating on the overall handling and feasibility of the HIV Qual Testing using the GeneXpert System. **d** Rating on how easy it was to prepare the test tubes (heel pricking, pipetting, mixing with buffer). **e** Rating on how easy it was in interpreting the test results

When asked on the frequency of using the Xpert user manual, all laboratory staff compared to only two clinic staff (18.2%) responded to using it often. None of the HCWs from primary health facility reported to using the manual often. Five (5/11) of secondary health facility staff and nine (9/13) primary health facility staff reported to have never used the manual (Fig. 2A).

Five and 6 of HCWs from primary and secondary health facilities respectively agreed or strongly agreed that the user manual was easy to understand, however three (3/8) and one (1/7) HCW from primary and secondary facilities respectively disagreed to this. All lab staff (4/4) agreed that the user manual was easy to understand (Fig. 2B).

### Gene Xpert handling

Test handling was evaluated at different steps including (i) heel pricking, pipetting and mixing of the blood sample with the test buffer, (ii) loading of the cartridge, (iii) starting of the Xpert analyser and the computer, and (iv) interpretation of the test result. Test handling was assessed according to the category of test experience of the clinic staff. Those that had tested > 20 samples were termed as “greater experienced” while those that performed 20 or less samples were termed as “lesser experienced”.

Over all, none of the staff rated gene Xpert handling as too complicated. However, three (3/15) and one (1/9) from lesser and greater experienced clinic staff respectively rated it as “not so easy”. All lab staff (4/4), about five of clinic staff with greater experience (5/9) and six (6/15) with lesser experience rated it as very easy (Fig. 2C).

All HCWs, regardless their level of experience in testing, reported that the preparatory stages of the testing tubes were easy or very easy (Fig. 2D).

All HCWs responded that interpretation of test results was easy or very easy. Of these, seven (7/15) of clinic staff with lesser test experience responded “easy” rather than “very easy”. None of the HCWs felt interpretation of results was not so easy or too complicated (Fig. 2E).

### Operation performance of the GeneXpert system and Xpert HIV-1 qual assay

**Fig. 3:**
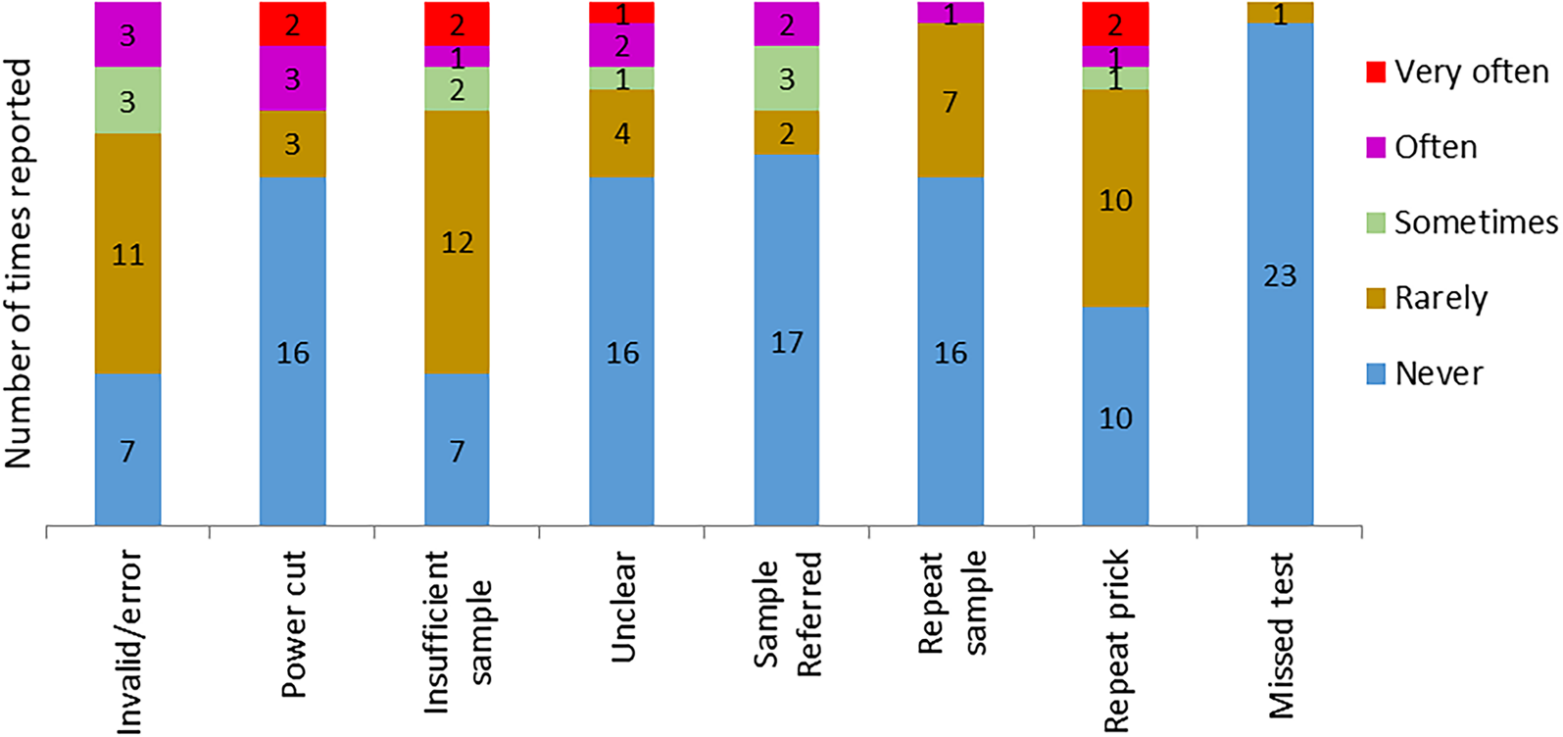
Operational Xpert PoC test performance at the health facilities

When asked how often they experienced different testing challenges and or other test mishaps, power cut (5/24), insufficient sample (3/24) and repeat prick (3/24) were reported as often or very often occurring by most HCWs. Despite the reported challenges, only one nurse reported that they rarely missed performing the test (Fig. 3).

### Overall impression on the use of the HIV-1 Qual PoC system in the public setting

**Fig. 4:**
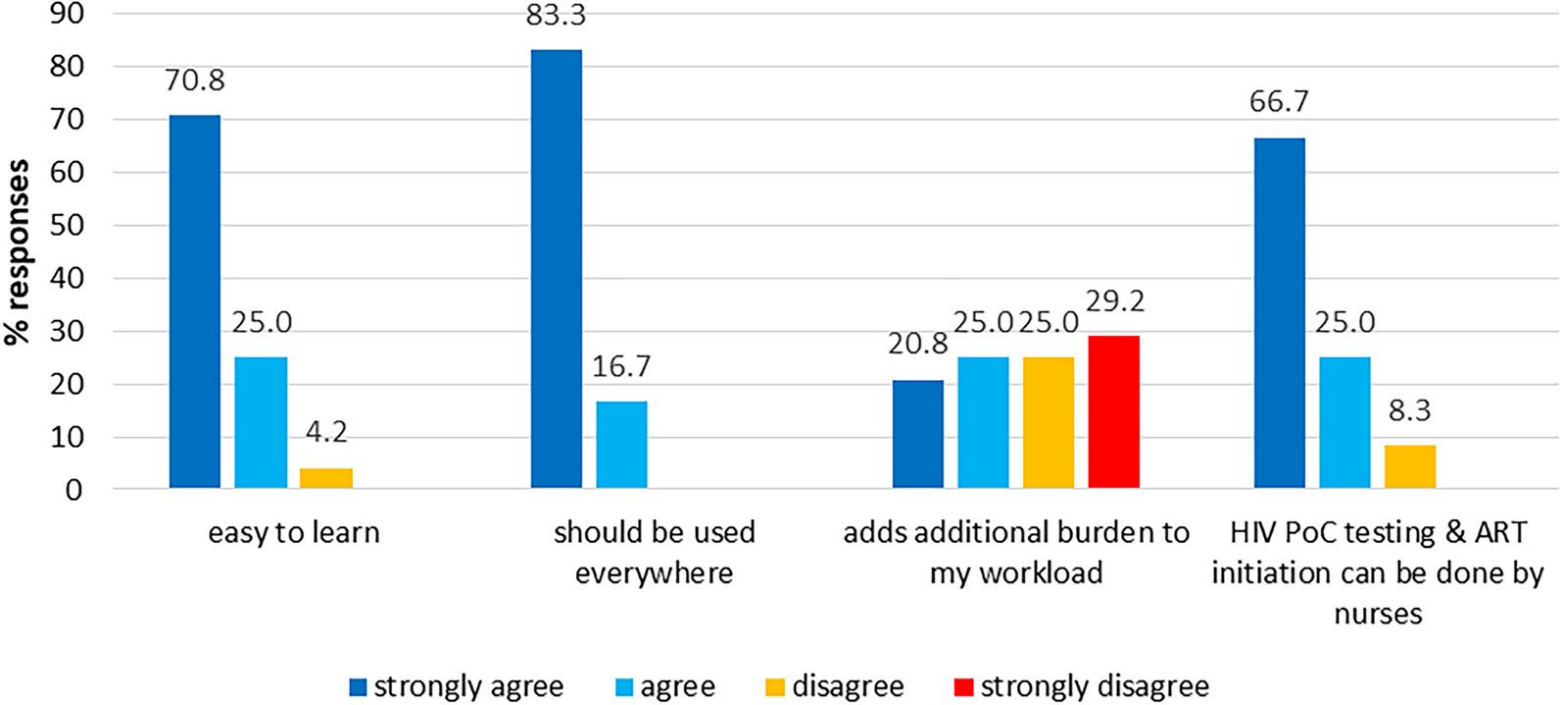
Overall impression on the use of PoC gene xpert system in public setting (N = 24)

All nurses except one reported that the HIV-1 Qual PoC was easy to learn for early infant diagnostics. One nurse who disagreed was from a primary health care centre, had acquired greater experience in PoC testing (20–50 tests performed), and had reported frequent problems with power cuts, frequent multiple heel pricks and referral of sample analysis to other health facilities. All nurses agreed or strongly agreed that HIV-1 Qual PoC testing should be generally used in clinical health care facilities. However, eleven (11/24) indicated that HIV-1 Qual PoC would add additional work burden to the routine workload which was independent whether they were affiliated to primary or secondary health care facilities or had acquired greater or lesser experience in PoC testing. The majority (22/24) of nurses reported that immediate ART initiation in the case of a positive HIV PoC result could be done by nurses applying to test & treat principles (Fig. 4).

## Discussion

This study has demonstrated that nurse based PoC HEID is feasible in resource constrained health care systems as a means to ensure timely test results and interventions for HIV exposed infants. From the main study, we demonstrated that nurses at different levels of the health care system, are capable of performing PoC testing reliably [9]. In this study, we were able to demonstrate a good overall impression and acceptability of PoC EID testing using Gene-Xpert among nurses based in primary health-care facilities. Both findings are similar to studies done in similar settings that assessed the performance and acceptability of PoC EID testing at nurse based primary health care clinics which showed it was both accurate, feasible and acceptable to have nurses perform PoC EID [12–14].

It was evident that, all laboratory staff found the two days training was adequate for meeting their skills performing tests. However, six (6/24) of the nurses found the training time not enough especially those from primary health facilities. All but two nurses from primary health facilities felt confident after the training. This may be explained by the numbers of nurses from primary health facilities that suggested more time to be provided for training and more samples to be run for training purposes. However, in the overall impression, almost all the nurses and all laboratory personnel agreed that this platform was easy to learn. Despite the perceptions of inadequacy of the training at the start, several on-job trainings and support were being provided continuously to increase the nurses’ experience and confidence in running the tests.

While all laboratory staff used the Xpert user manual, only two nurses from secondary health facility did use the manual. Those nurses from primary health facility were even less likely to use the manual for reference. Though reasons for not using the manual were not assessed, some challenges such as language barrier may have contributed to this. The manual was in English language, which is not as comfortable as Swahili especially in lower level facilities. The manual did however use simple, easy to understand wording rather than technical terms making it easy for those who reported to have used it often. This highlights the consideration for language translation in development of manuals and job-aides especially in settings as these.

All of the HCWs agreed that HIV-1 Qual PoC testing should be generally used in clinical health care facilities. This was similarly demonstrated in a study done in South Africa, where acceptability of PoC testing was high among nurses at the facilities [15]. Half (11/24) of the nurses did however point out that HIV-1 Qual PoC would add a burden to their routine workload. This was despite the compensation they received for every participant that was attended. In programmatic settings, adopting nurse based PoC testing, would require designating this duty to nurses who are less occupied with other activities at the clinic as well as prioritizing testing at the clinic for high risk mother-infant pairs that require immediate decisions on ART initiation and or enhanced prophylaxis.

Due to the unreliable power supply in most rural and semi urban settings, operationalization of PoC platforms would require a sustainable back up such as solar power as a means to ensure continued testing even in the event of power outages. This operational feature is important in resource constrained settings and PoC platforms with the ability to store charge or use a backup power supply have shown to function well even at community levels [16].

Our study was limited by the small sample size of health care workers involved in the assessment due to the design of the primary study that involved only a number of HCWs in the selected facilities. Also, since the Xpert HIV-1 qual assay was still an investigational assay not yet prequalified by the WHO, test results were not used for medical decision making but rather based on the conversational platforms (Taqman) at the centralized laboratory [17]. Thus, we were unable to assess the impact of Xpert HIV-1 qual on immediate treatment initiation, or infant’s health outcomes. This study operational feasibility assessment was done in clinical trial settings rather than programmatic settings, however, the results highlight on the possible opportunity of involving nurses in performance of PoC EID testing platforms.

In conclusion, nurses at primary health facilities demonstrated very good perception and experiences of using Xpert HIV-1 qual platform for EID testing. With appropriate training, targeted task distribution at the clinics, continual supportive supervision and power back up mechanisms, it is possible to have efficient nurse based PoC EID testing. This decentralization of tests will ensure effective implementation of birth testing, timely availability of results and initiation of follow up with prompt clinical decisions for prophylaxis and treatment [11, 13]. Nurse based PoC tests are not meant to replace the conventional testing that is currently available, but rather supplement it, especially in the most remote areas where, due to structural challenges, are more likely to have longer TATs for HEID results.

### Supplementary Information

**Additional file 1. **Nurse/User interview questionnaire.

## Data Availability

The datasets analysed during the study are available from the corresponding author on reasonable request.

## Abbreviations

HIV: Human Immunodeficiency Virus
PoC: Point of Care
EID: Early infant diagnosis
HEID: HIV early infant diagnosis
HCWs: Health care workers
ART: Anti-retroviral treatment
CTC: Care and Treatment Centre
PVHT: Prevention of vertical HIV transmission
NAT: Nucleic acid testing
PNP: Postnatal prophylaxis
E-PNP: Enhanced postnatal prophylaxis
DBS: Dry blood spot
WHO: World Health Organization
NIMR: National Institute for Medical Research
MMRC: Mbeya Medical Research Centre
MZRH: Mbeya Zonal Referral Hospital
MRRH: Mbeya Regional Referral Hospital
