# Supplementary material for: Xpert HIV-1 qual point-of-care testing for HIV early infant diagnosis in Tanzania: experiences and perceptions of health care workers in a 2016 study
Source: AIDS Res Ther. 2024 May 16;21:33. doi: 10.1186/s12981-024-00619-2 (PMC11097447; doi:10.1186/s12981-024-00619-2)
Supplement: Supplementary file 1 — Additional file 1. Nurse/User interview questionnaire. [file 12981_2024_619_MOESM1_ESM.pdf]

Xpert HIV-1 Qual: Nurse/User interview

Site information

Name of the site<br>O Igawilo<br>O META<br>O Kiwanjampaka<br>O Ruanda<br>O Regional	Numbers of nurses/midwives trained on the Xpert POC: _______<br>Degree: O nurse degree O nurse certificate O nurse diploma O clinical officer<br>Gender: O male O female<br>Age: ______________

**Training**				
Were you trained on how to use the GeneXpert for HIV HIV-1 Qual testing	☐ yes ☐ no			
	Strongly Agree	***Agree***	***Disagree***	***Strongly Disagree***
The level of the Xpert PoC test training was adequate to my prior knowledge and abilities (neither too easy nor to difficult).	O	O	O	O
There was enough time to acquire the intended skills.	O	O	O	O
I had enough opportunity to run Xpert test samples for practical training	O	O	O	O
After the training I felt confident using the Xpert PoC test	O	O	O	O
How many test runs for the training did you perform	☐ 1 ☐ 2 ☐ 3 ☐ >3			
**Xpert user manual**				
Did you have access to the GeneXpert user manual	☐ yes ☐ no			
Did you use the GeneXpert user manual	☐ Often ☐ Sometimes ☐ Rarely ☐ Never			
	Strongly agree	***Agree***	***Disagree***	***Strongly Disagree***
The GeneXpert user manual is easy to understand	O	O	O	O
The GeneXpert user manual is helpful if there is a problem	O	O	O	O
**GeneXpert handling**				
About how many times have you used the Xpert PoC test during the study	☐ <10 times; ☐ 10 to 20 times; ☐ 20 to 50 times; ☐ >50 times			
	Very easy	***Easy***	***Not so easy***	***Too complicated***
How would you rate/grade the overall handling and feasibility of the HIV Qual Testing using the GeneXpert System	O	O	O	O
Specifically how would you rate/grade the following steps				
- preparation of the test tubes (prick, pipetting, mixing with buffer)	O	O	O	O

- loading of the cartridge	O	O	O	O	
- starting the test on the Xpert analyser and the computer	O	O	O	O	
- interpretation of the test result	O	O	O	O	
	Very often	***Often***	***Sometimes***	***Rarely***	***Never***
Did you experience invalid test results or error messages	O	O	O	O	O
If there were invalid results or error messages, how often were the following reasons					
- power cut	O	O	O	O	O
- not enough sample loaded into the cartridge	O	O	O	O	O
- did not know what was the problem	O	O	O	O	O
- others, indicate:_____________________________	O	O	O	O	O
What did you do usually do when there was an invalid result or error message					
- I referred the sample to another clinic for testing	O	O	O	O	O
- I referred the mother and child to another clinic for testing	O	O	O	O	O
- I repeated the test using the same sample	O	O	O	O	O
- I repeated the test and had to re-prick the infant	O	O	O	O	O
- I skipped the HIV testing completely	O	O	O	O	O
- others, indicate:_____________________________	O	O	O	O	O
**Communication of test results to the mother**	***Strongly agree***	***Agree***	***Disagree***	***Strongly Disagree***	
In general the HIV test results of the child can be provided to the mother at the same day	O	O	O	O	
Mothers are happy to receive the HIV test results for their child immediately	O	O	O	O	
How long does a mother need to stay at the clinic to receive the HIV test result of the child	☐ 2 hours; ☐ 3 hours; ☐ 4 hours; ☐ 6 hours; ☐ >6 hours				

**Overall impression**	***Strongly agree***	***Agree***	***Disagree***	***Strongly Disagree***
The Xpert HIV-1 Qual PoC testing is easy to learn?	O	O	O	O
I think that HIV-1 Qual PoC testing by nurses/midwives should be used everywhere	O	O	O	O
The Xpert HIV-1 Qual PoC testing adds significant additional burden to my workload	O	O	O	O
In infant with HIV-1 Qual PoC positive test results infant ART could be immediately initiated by nurses	O	O	O	O
General comments, remarks, suggestions				

Thank you !
